# The relation between serum uric acid levels and diabetic peripheral neuropathy in type 2 diabetes in Guilan, north of Iran

**DOI:** 10.1186/s12902-022-00952-5

**Published:** 2022-02-12

**Authors:** Haniye Sadat Fayazi, Maryam Yaseri, Seyyede Sahere Mortazavi, Zahra Sharifhassan, Ali-Sina Assadinia

**Affiliations:** 1 Department of Internal Medicine, School of Medicine, Razi Hospital, Guilan University of Medical Sciences, Rasht, Iran; 2 Razi Clinical Research Development Unit, Guilan University of Medical Sciences, Rasht, Iran

**Keywords:** Type 2 diabetes, Diabetic polyneuropathy, Uric acid, Cardiovascular disease

## Abstract

**Background:**

Diabetic peripheral neuropathy (DPN) is one of the most common chronic microvascular complications in type 2 diabetes mellitus (T2DM). Hence, the present study aimed to investigate the association between Serum Uric Acid (SUA) levels and diabetic peripheral polyneuropathy in patients with type 2 diabetes.

**Methods:**

We performed this case–control study during 2019–2020 on individuals with diabetes referring to the Razi clinic of Rasht, in the north of Iran. Polyneuropathy in patients was assessed based on the Neuropathy Disability Score (NDS), Diabetic neuropathy symptom score (DNS) scoring system, and electromyography (EMG)/nerve conduction velocity (NCV). The inclusion criterion for the control group was normal EMG/NCV. Then, the patients were assessed for SUA level and also laboratory results.

**Results:**

In total, 230 patients with type 2 diabetes were examined. The mean SUA level in the DPN group was significantly higher compared to the control group (6.72 ± 1.75 vs. 4.57 ± 1.49 mg/dL). With increasing the SUA, the odds of developing neuropathy increased by 2.2 times (OR = 2.2). The risk factors for diabetic polyneuropathy included gender (male) (OR = 0.347), SBP (OR = 1.1), retinopathy (OR = 3.29), and microalbuminuria (OR = 4.44). The chance of developing polyneuropathy in patients with retinopathy was 3.3 times higher than in the control group, it was 4.4 times in microalbuminuria patients.

**Conclusion:**

Elevated SUA level increased the chance of developing peripheral polyneuropathy in a person with type 2 diabetes. SUA levels higher than 5.25 mg / dL expose a person with type 2 diabetes to developing peripheral polyneuropathy.

## Background

Type 2 diabetes mellitus (T2DM) is associated with long-term complications resulting in cardiovascular diseases, renal failure, and nephropathy [1, 2]. It has increased health problems and become a worldwide concern [3, 4]. Type 2 DM involves 90–95% of diabetics and is more prevalent among the elderly [5, 6]. Diabetic Peripheral Neuropathy (DPN) is one of the most common chronic microvascular complications in progressing of T2DM to diabetic foot ulcer [6]. It frequently leads to amputation and/or disability and death. DPN accounts for 30–50% of patients with diabetic leg and foot ulcers [7]. The main related factors included age > 40 years, obesity, and hypertension [8–10]. However, its prevalence and the risk factors on a global scale remain unclear especially in the low- and middle-income countries. Previous research revealed that hyperuricemia with hyperglycemia, insulin resistance, dyslipidemia, and metabolic syndrome, all are involved in the development of diabetic neuropathy [11–13].

According to the reports in 2020, serum uric acid (SUA) level ≥ 7.3 mg/dL was found to be associated with an increase in peripheral neuropathy [14]. The high SUA level was related to the incidence of macro and microvascular complications in patients with DM. An elevated level of SUA in T2DM was associated with metabolic syndrome and insulin resistance [15], as well as with a higher risk of developing diabetic polyneuropathy [16]. Previous research identified SUA as a sign of oxidative stress from hyperuricemia, which can cause insulin resistance, diabetes, and cardiovascular disease [17]. However, SUA -lowering therapy has a positive effect on reducing the incidence of T2DM and insulin resistance [18].

The presence of DPN affects the quality of life and increases death from cardiovascular disease. Unfortunately, there is no certain therapy definitively eliminating the symptoms of diabetic neuropathy. Therefore, identifying the relationship between diabetic polyneuropathy and its associated risk factors is essential to determine appropriate treatment and provide prevention and screening measurements. Acceding to the previous studies, there is a significant relationship between SUA levels and diabetic peripheral polyneuropathy. However, there is still no agreement among them, and further studies are recommended in this area. This study aimed to investigate the relationship between SUA levels and diabetic peripheral polyneuropathy in patients with type 2 diabetes referring to RAZI Clinic in Rasht during 2019–2020.

## Material & method

### Patients and design

In this case–control study, individuals with diabetes referring to Razi Clinic in Rasht, the north of Iran, during 2019–2020 were studied. The patients were included by the Consecutive sampling method and based on diabetic peripheral polyneuropathy. They were evaluated in two groups of patients with and without diabetic peripheral polyneuropathy (control group). The two groups were matched in terms of age, sex, BMI, and disease duration of diabetes.

### Inclusion and exclusion criteria

All individuals with diabetes were included in this study. The exclusion criteria included the age of less than 18 or over 75 years; underlying disease of gout, peripheral arterial disease, chronic kidney disease (creatinine > 2 mg / dL or eGFR < 30 min), chronic or acute infections, and blood disorders; taking drugs affecting the serum level of SUA such as diuretics, cyclosporine, allopurinol, estrogen, and cytotoxic drugs; and the presence of factors resulting in neuropathy such as vitamin B12 deficiency, alcohol abuse, cancer, and peripheral nerve damage.

### Assessment tools and measurements

Polyneuropathy in patients was examined by the Neuropathy Disability Score (NDS), Diabetic neuropathy symptom score (DNS) scoring system, and electromyography (EMG)/nerve conduction velocity (NCV). The person with type 2 diabetes was initially evaluated according to the DNS scoring system through four sign points including unsteadiness in walking, numbness, burning/aching pain or tenderness in the lower limbs, and prickling sensation. Patients who did not score were considered as the control group (without peripheral polyneuropathy). The patients with at least one score were evaluated for the NDS scoring system based on the signs of neuropathy in the ankle reflex (Achilles), the pinprick, temperature sensation at both sides of the great toes, and vibration sensation (128-Hz tuning fork). The patients with an NDS score ≥ 6, were diagnosed with diabetic peripheral polyneuropathy. The patients with a score < 6 and with at least one of the four points of DNS, were subjected to EMG / NCV. By the abnormal EMG / NCV, the patients were diagnosed with DPN; otherwise included in the control group. In this study, demographic information was also recorded including age, gender, smoking status (one per day for at least two years), history of kidney disease or hypertension. Information on the SUA levels was evaluated along with LDL, HDL, Total Cholesterol, Triglyceride, HB1AC, and serum creatinine in two groups of patients. SUA status was considered based on the study of Xiaopu Lin et al. [19] in four categories (> 5, 5.1–7, 7.1–9, > 9). Microalbuminuria was examined based on the latest urine random test, which confirmed the ratio of albumin to urinary creatinine between 30 mg/g microalbuminuria.

### Statistical analysis

An Independent T-test was used to analyze the data and compare the two groups. A chi-squared test was utilized to compare the frequency distribution of SUA levels in the two groups. Pearson and Spearman correlation coefficient was used to determine the correlation of SUA level with the studied quantitative variables (systolic pressure, diastolic pressure, lipid profile, and serum creatinine). Ultimately, the logistic regression model was utilized to determine the relationship between SUA levels and diabetic polyneuropathy after adjusting for the effects of individual and social interfering variables. The significance level of tests in this study was considered as *P* < 0.05. All statistical analyses were performed using SPSS 16 (IBM Corp., USA). Statistical significance level was considered as a *P*-value of < 0.05.

Also, CBC test was measured at Razi Hospital by (Sysmex XK 21-N, Germany) and creatinine and albumin levels were measured by (HITACHI autoanalyzer 717, Tokyo, Japan).

## Results

In the present study, 230 people with type 2 diabetes were studied in two groups of patients with and without peripheral diabetic polyneuropathy. The mean age of patients was 57.4 ± 8.1 years and ranged from 36 to 74 years. Of the total patients, 69.6% were females. The mean BMI was 28.1 ± 4.6. The patients with overweight were 38.7%, obese 22.6%, and very obese were 10%. There was no significant difference between the two groups regarding age (*P* = 0.669), gender (*P* = 0.667), and BMI (*P* = 0.427). The diabetes duration in patients with peripheral diabetic polyneuropathy was 12.9 ± 7.9 years and it was 10.3 ± 6.13 years in those without (*p* = 0.004). Other patient information in the two groups is presented in Table 1.

**Table 1:** Comparing patients ‘characteristics between DPN and control group

Variable	level	DPN		Control		Total		*P*-value
		n	%	n	%	n	%	
Age (y)	< 50	20	17.4	25	21.7	45	19.6	0.669
	51–60	53	46.1	48	41.7	101	43.9	
	> 60	42	36.5	42	36.5	84	36.5	
Gender	Female	78	67.8	82	71.3	160	69.6	0.667
	Male	37	32.2	33	28.7	70	30.4	
BMI	Thin	3	2.6	5	4.3	8	3.5	0.427
	Normal	27	23.5	31	27	58	25.2	
	overweight	47	40.9	42	36.5	89	38.7	
	Obese	23	20.0	29	25.2	52	22.6	
	Very obese	15	13.0	8	7	23	10	
Smoking	yes	20	17.4	23	20	43	18.7	0.61
	no	95	82.6	92	80	187	81.3	
Retinopathy	yes	66	57.4	25	21.7	91	39.6	< 0.001
	no	49	42.6	90	78.3	139	60.4	
microalbuminuria	yes	79	68.7	27	23.5	106	46.1	< 0.001
	no	36	31.3	88	76.5	124	53.9	
Diabetes duration (y)	< 2	6	5.2	12	10.4	18	7.8	0.456
	3–5	16	13.9	16	13.9	32	13.9	
	6–10	30	26.1	32	27.8	62	27	
	> 10	63	54.8	55	47.8	118	51.3	
SBP (mmHg)	**-**	136.78 ± 16.57		132.52 ± 16.27		134.65 ± 16.52		0.05
DBP (mmHg)	**-**	80.57 ± 10.45		79.87 ± 10.85		80.22 ± 10.63		0.621
FBS (mg/dl)	**-**	206.59 ± 76.60		176.34 ± 63.14		191.47 ± 71.66		0.001^*^
HbA1c (%)	**-**	8.42 ± 1.09		7.45 ± 1.14		7.94 ± 1.21		< 0.001
Creatinine (mg/dl)	**-**	0.99 ± 0.16		0.89 ± 0.15		0.94 ± 0.16		< 0.001
Cholesterol (mg/dl)	**-**	178.70 ± 46.85		155.87 ± 40.62		167.28 ± 45.22		< 0.001
LDL (mg/dl)	**-**	102.09 ± 35.87		88.17 ± 34.06		95.3 ± 35.59		0.003^*^
HDL (mg/dl)	**-**	39.72 ± 7.56		39.71 ± 7.5		39.72 ± 7.5		0.05
Triglyceride (mg/dl)	**-**	203.23 ± 84.63		170.27 ± 63.97		186.75 ± 86.65		0.621

*BMI* Body mass index, *SBP* Systolic blood pressure, *DBP* Deltamethrin based pesticide, *FBS* Fasting blood sugar, *HDL* High-density lipoprotein, *LDL* Low-density lipoprotein

In total, 18.7% of the patients was a smoker and the frequency of smoking was not significantly different between the two groups. Diabetic retinopathy was found in 39.6% of the patients. In the DPN group, it was seen in 57.4%, which was approximately 5.2 times higher than the control group (21.7%) (*P* < 0.001). Microalbuminuria was observed in 46.1% of patients. In the DPN group, it was observed in 68.7%, which was almost 3 times higher than the control group (23.5%) (*P* < 0.001). Systolic blood pressure (*P* = 0.05), FBS (*P* = 0.001), HbA1c (*P* < 0.001), was significantly higher in the DPN group compared to the control group.

Table 2 represents the results of comparing the SUA between the two groups. The mean SUA in the DPN group was significantly higher than the control group (6.72 ± 1.75 mg/dL vs. 4.57 ± 1.49 mg/dL) (*P* < 0.001). Moreover, the level of SUA was significantly higher in the DPN group (11.3% vs. 1.7%).

**Table 2:** Comparing Serum uric acid levels between the DPN group and control group

Variable	LEVEL	DPN		Control		*P*-value
		n	%	n	%	
Serum uric acid level (mg/dl)	≤ 5	22	19.1	88	76.5	< 0.001
	5.1–7	51	44.3	19	16.5	
	7.1–9	29	25.2	6	5.2	
	> 9	13	11.3	2	1.7	
Serum uric acid (Mean ± SD) (mg/dl)	-	6.72 ± 1.75		4.57 ± 1.49		< 0.001

The results of logistic regression for the relationship between SUA and diabetic peripheral polyneuropathy adjusted for other variables in the model are reported in Table 3. It revealed that SUA was significantly associated with neuropathy in both adjusted (*P* < 0.001, OR = 2.2) and the non-adjusted models (*P* < 0.001, OR = 2.3). With increasing the SUA, the probabilities of developing neuropathy increased by 2.2 times (OR = 2.2). Moreover, the results indicated that male patients were less likely to develop polyneuropathy compared to females (*P* = 0.022, OR = 0.347) so that women were 2.9 times at risk of polyneuropathy. Systolic blood pressure (*P* = 0.005, OR = 1.1) and Retinopathy (*P* = 0.003, OR = 3.29) increased the risk of polyneuropathy. A history of hypertension reduced the risk of developing polyneuropathy (*P* = 0.002, OR = 0.233). The microalbuminuria incremented the risk of developing polyneuropathy by 4.4 times (*P* < 0.001, OR = 4.44).

**Table 3:** Results of logistic regression in relation between DPN and Serum uric acid after adjusting for other factors

Model	Variable	B	S.E	*P*-value	OR	95% C.I. for OR	
						Lower	Upper
**Unadjusted**	Serum uric acid	0.821	0.147	0.000	2.272	1.702	3.033
	Age	0.001	0.028	0.977	1.001	0.948	1.056
	Gender	-0.790	0.673	0.240	0.454	0.121	1.697
	BMI	-0.070	0.049	0.159	0.933	0.846	1.028
	SBP	0.051	0.019	0.006	1.052	1.014	1.091
	DBP	-0.057	0.028	0.040	0.945	0.895	0.997
	Smoking	-0.749	0.727	0.303	0.473	0.114	1.967
	Retinopathy	1.119	0.434	0.010	3.062	1.309	7.164
	Diabetes duration (y)	-0.005	0.032	0.880	0.995	0.934	1.060
	Hypertension history	-1.416	0.513	0.006	0.243	0.089	0.664
	Cardiovascular history	0.683	0.492	0.165	1.980	0.755	5.191
	FBS (mg/dl)	0.003	0.003	0.435	1.003	0.996	1.009
	HbA1c	0.363	0.209	0.082	1.438	0.955	2.165
	Creatinine (mg/dl)	-0.117	1.763	0.947	0.889	0.028	28.157
	Microalbuminuria	1.438	0.550	0.009	4.213	1.432	12.390
	Cholesterol (mg/dl)	0.012	0.018	0.515	1.012	0.977	1.048
	LDL (mg/dl)	-0.013	0.019	0.481	0.987	0.951	1.024
	HDL (mg/dl)	-0.012	0.035	0.725	0.988	0.923	1.058
	Triglyceride (mg/dl)	-0.003	0.004	0.395	0.997	0.990	1.004
**Adjusted**	Serum uric acid (mg/dl)	0.768	0.130	0.000^*^	2.155	1.671	2.781
	Gender (male vs. female)	-1.059	.463	0.022^*^	0.347	0.140	0.859
	SBP	0.049	0.018	0.005^*^	1.051	1.015	1.088
	Retinopathy	1.190	0.404	0.003^*^	3.288	1.491	7.252
	Hypertension history	-1.457	0.464	0.002^*^	0.233	0.094	0.578
	HbA1c	0.341	0.177	0.054	1.406	.994	1.989
	Microalbuminuria	1.491	0.410	0.001^*^	4.440	1.988	9.920

*BMI* Body mass index, *SBP* Systolic blood pressure, *DBP* Deltamethrin based pesticide, *FBS* Fasting blood sugar, *HDL* High-density lipoprotein, *LDL* Low-density lipoprotein

Table 4 presents the results of comparing SUA levels in terms of patients’ demographic per groups. In the DPN group, SUA level was affected by an older age (*P* < 0.003), male gender (*P* < 0.009), high BMI (very obese) (*P* < 0.008), retinopathy (*P* < 0.004) and microalbuminuria, hypertension history (*P* < 0.005), and heart disease. Furthermore, a significant positive association was found between SUA with the quantitative variables in the DPN group and age (*r* = 0.282, *P* = 0.002), BMI (r = 0.194, *P* = 0.38), diabetes duration (r = 0.091, *P* = 0.334), diastolic blood pressure (*r* = 0.189, *P* = 0.043) and creatinine (*r* = 0.206, *P* = 0.027).

**Table 4:** Association between patients ‘characteristics and uric acid in DPN group and control group

**Variable**	**level**	**Control**^a^		**DPN**^b^		***P*-value**^a^	***P*-value**^b^
		**Mean**	**SD**	**Mean**	**SD**		
Age (y)	< 50	4.51	1.31	6.08	1.43	0.312	0.003^*^
	51–60	4.37	1.32	6.40	1.67		
	> 60	4.85	1.75	7.42	1.79		
Gender	Female	4.20	1.10	6.42	1.55	< 0.001	0.009^*^
	Male	5.50	1.90	7.34	2.00		
BMI	Thin	5.26	1.86	6.50	2.43	0.112	0.008^*^
	Normal	4.06	0.90	6.96	200		
	overweight	4.67	1.66	6.09	1.59		
	Obese	4.64	1.63	7.01	1.46		
	Very obese	5.40	1.29	7.83	1.46		
Smoking	yes	5.33	2.09	7.00	2.16	0.006^*^	0.438
Retinopathy	yes	4.83	1.95	6.84	1.72	0.332	0.004^*^
Diabetes duration (y)	< 2	4.14	1.21	6.85	2.42	0.176	0.919
	3–5	4.26	1.07	6.60	2.21		
	6–10	4.35	1.36	6.56	1.85		
	> 10	4.89	1.68	6.81	1.54		
Hypertension history	yes	4.75	1.61	7.05	1.56	0.061	0.005^*^
Cardiovascular history	yes	4.51	1.44	6.49	1.85	0.832	0.350
Microalbuminuria	yes	4.85	1.88	6.86	1.79	0.279	0.211
		**R**	***p*-value**	**R**	***p*-value**		
SBP	-	0.126	0.178	0.160	0.087		
DBP	-	0.189	0.043^*^	0.161	0.085		
FBS (mg/dl)	-	-0.064	0.498	0.019	0.840		
HbA1c	-	0.138	0.141	0.119	0.207		
Creatinine (mg/dl)	-	0.206	0.027^*^	0.251	0.007^*^		
Cholesterol (mg/dl)	-	-0.015	0.871	-0.014	0.885		
LDL (mg/dl)	-	-0.029	0.757	-0.019	0.840		
HDL(mg/dl)	-	-0.150	0.109	-0.126	0.178		
Triglyceride (mg/dl)	-	0.082	0.381	0.044	0.643		

*BMI* Body mass index, *SBP* Systolic blood pressure, *DBP* Deltamethrin based pesticide, *FBS* Fasting blood sugar, *HDL* High-density lipoprotein, *LDL* Low-density lipoprotein

**Fig. 1:**
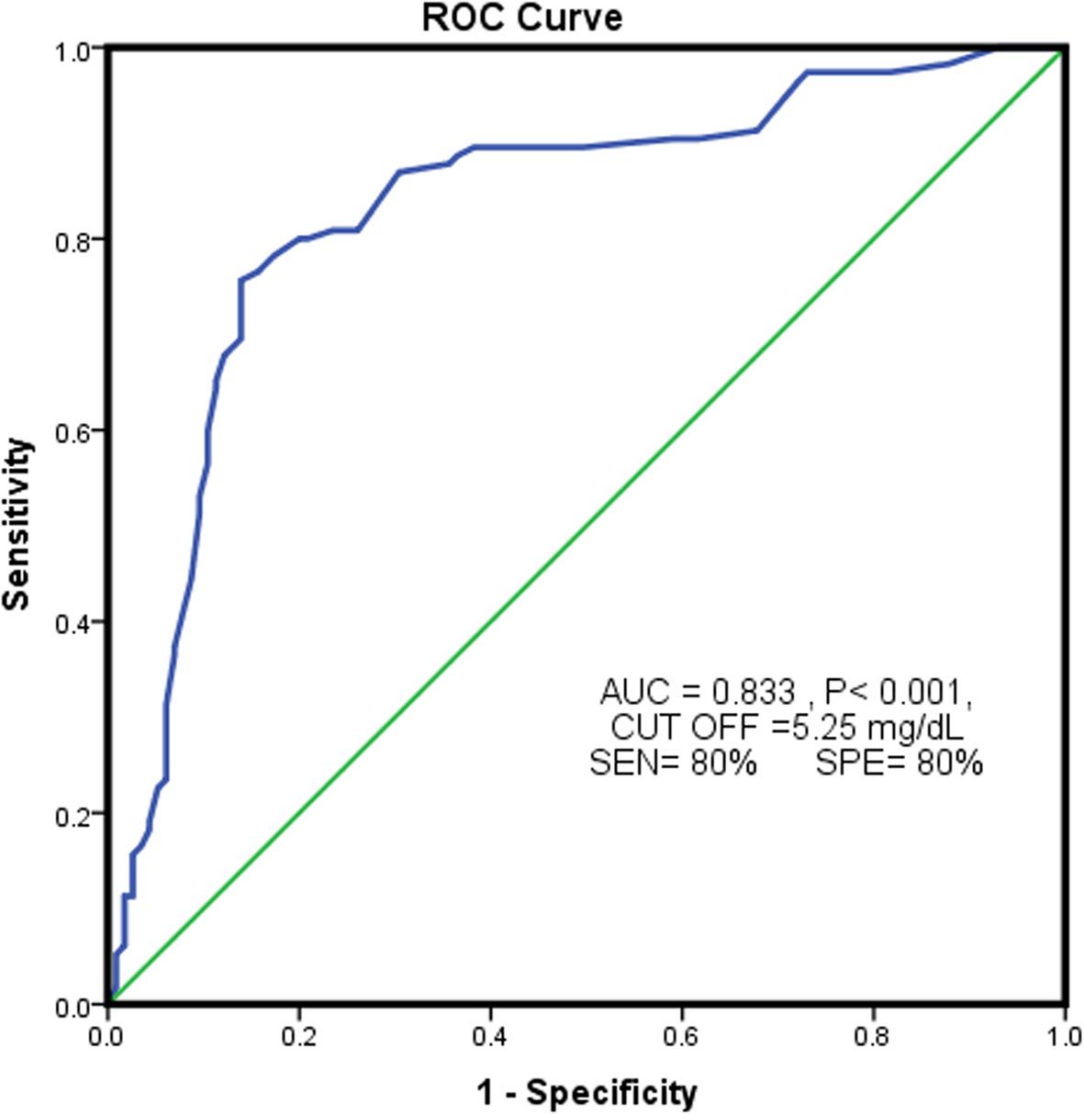
ROC curve to predict uric acid level for diabetic polyneuropathy

Regarding the significant relationship between SUA and DPN, the ROC curve was used to predict SUA for polyneuropathy in a person with type 2 diabetes (Fig. 1). The area under the level of SUA for predicting diabetic neuropathy was statistically significant (*P* < 0.001, AUC = 0.833). Considering sensitivity and specificity values, the best cut-off point for SUA was 5.25 mg / dL, for which sensitivity and specificity were 80%. The people with type 2 diabetes with SUA levels > 5.25 mg/dL were at a higher risk of polyneuropathy.

## Discussion

DPN is one of the most common chronic microvascular complications in T2DM. The high SUA level is associated with an increase in DPN. Moreover, reduced mobility and sensation and increased disability can be caused by DPN. Also, recent reports declare that younger adults with a high level of SUA are at a higher risk of developing type 2 diabetes independent of other well-known risk factors such as BMI, physical activity level, age, hypertension, alcohol consumption, smoking, levels of glucose, creatinine, triglycerides, and cholesterol [20, 21].

In this study, we aimed to investigate the relationship between SUA levels and diabetic peripheral polyneuropathy in patients with type 2 diabetes referring to RAZI Clinic in Rasht during 2019–2020. Our results revealed that the mean SUA in the DPN group was significantly higher than the control group (6.72 mg/dL vs. 4.57 mg/dL). Similar results were reported in studies by Papanas et al. [22] and Xiaopu Lin [19].

Moreover, in the present study, the high level of SUA was significantly more in the DPN group (11.3% vs. 1.7%). Regarding the SUA classification in the study by Xiaopu Lin et al. (20), its frequency percentage in DPN patients was higher than the control group (level of > 9: 11.3% vs. 1.7; the level of 7.1- 9: 25.2% vs. 5.2%; the level of 7–5.1: 44.3% vs. 16.5%). Also, according to the results obtained from the ROC curve in the present study, the patients with high levels of SUA (> 5.25 mg /dL) were at a higher risk of developing polyneuropathy. In the study of Xiaopu Lin et al. [19], SUA levels > 7.8 mg /dL were associated with an increased incidence of polyneuropathy. Wisit Kaewput et al., (2002) found that an SUA level ≥ 7.3 mg/dL was found to be associated with an increased peripheral neuropathy compared with an SUA level < 4.4 mg/Dl (OR,1.54) [14].

SUA was significantly associated with neuropathy based on the results of logistic regression for the relationship between SUA and diabetic peripheral polyneuropathy adjusted for other variables in the model. With increasing the SUA, the odds of developing neuropathy increased by 2.2 times (OR = 2.2). Considering our results, it should be noted that the studied population included high-risk individuals, which could justify the results. In this regard, the results of previous studies pointed to the high levels of SUA and its association with type 2 diabetes [20].

Smith et al. revealed that Metabolic Syndrome features increased in 219 individuals with idiopathic neuropathy in comparison with controls without neuropathy [23].

According to previous studies from 2011 to 2014, increased SUA levels were associated with the risk of diabetic polyneuropathy. They may also be affected by diabetic polyneuropathy so that hyperuricemia increases the chance of developing diabetic peripheral polyneuropathy by about 2.83 [16]. These studies revealed that SUA was significantly higher in diabetic polyneuropathy [22, 24]. Regarding the conflict results, more studies have recently been conducted to investigate the relationship between SUA and DPN. Lin et al., (2017) studied 200 people with type 2 diabetes and found that systolic and SUA were different between the two groups with and without DPN [19]. However, a study by Laura Gaita et al., (2019), on 133 individuals with type 2 diabetes showed that there was no association between hyperuricemia and diabetic polyneuropathy [25]. In other results of the present study, gender (OR = 0.347), systolic blood pressure (OR = 1.1), retinopathy (OR = 3.29), and microalbuminuria (OR = 4.44) were identified as risk factors for polyneuropathy. Male patients were less likely to develop polyneuropathy compared to females (OR = 0.347) so that women were 2.9 times at risk of polyneuropathy. Abraham et al., (2017) examined the clinical records, neurological and electrophysiological examinations, and laboratory findings of 115 DPN patients and comparing them with 23 controls and 38 people with type 2 diabetes without DPN. They found a positive correlation between SUA levels, male gender, and hypertension [26].

In the present study, retinopathy was observed in 57.2% of patients with DPN. Moreover, the chance of developing polyneuropathy in patients with retinopathy was about 3.3 times higher than in the control group. In the study conducted by Papanas et al., [22], the frequency percent of retinopathy was 62.5% in DPN patients and 48.5% in the control group. However, no significant difference was reported between the two groups. Hou et al., (2020) indicated that the higher levels of SUA (Q3 and Q4) were associated with greater risk for diabetic retinopathy, compared with the lower level (Q1) (odds ratio [OR]: 3.056, 95% confidence interval [CI]: 1.506–6.198; OR: 3.417, 95% CI: 1.635–7.139, respectively) [27].

In the present study, the frequency of microalbuminuria in DPN was 68.7% and the chance of developing microalbuminuria was 4.4 times higher in patients than in the control group. Papanas et al., [22] found no statistically significant difference between the two study groups in terms of microalbuminuria. Hou et al., (2020) revealed that the higher levels of SUA (Q2, Q3, and Q4) were associated with greater risk for albuminuria (OR: 2.418, 95% CI: 1.059–5.522; OR: 7.233, 95% CI: 3.145–16.635; and OR: 8.911, 95% CI: 3.755–21.147, respectively) [27]. In the present study, the mean systolic blood pressure in patients with DPN was higher than in the control group. Similar results were reported in the study by Lin et al. [19]. The current study encountered some limitations. First, since this was a single-center study, the generalizability of the results to other regions may require further investigation. Second, some patients withdrew from the study.

## Conclusion

In the present study, the mean serum level of SUA in a person with type 2 diabetes with peripheral polyneuropathy was significantly higher than the control group. With increasing one unit of SUA, the chance of developing peripheral polyneuropathy in patients increased about 2.2 times. The higher level of SUA level above 5.25 mg / dL exposed the patient to peripheral polyneuropathy. Further prospective cohort studies are required to evaluate the effect of SUA levels on diabetic peripheral polyneuropathy and disease progression.

## Data Availability

The datasets used and/or analyzed during the current study are available from the corresponding author on reasonable request.

## Abbreviations

BMI: Body mass index
CI: Confidence interval
DPN: Diabetic Peripheral Neuropathy
NDS: Neuropathy Disability Score
DNS: Diabetic neuropathy symptom score
EMG: Electromyography
NCV: Nerve conduction velocity
SUA: Serum uric acid
T2DM: Type 2 diabetes mellitus
OR: Odds ratio
